# *GJB2 c*.35del variant up-regulates *GJA1* gene expression and affects differentiation of human stem cells

**DOI:** 10.1590/1678-4685-GMB-2023-0170

**Published:** 2024-04-15

**Authors:** Ana Carla Batissoco, Dayane Bernardino Cruz, Thiago Geronimo Pires Alegria, Gerson Kobayashi, Jeanne Oiticica, Luis Eduardo Soares, Maria Rita Passos-Bueno, Luciana Amaral Haddad, Regina Célia Mingroni

**Affiliations:** 1 Universidade de São Paulo (USP), Faculdade de Medicina (FM), Hospital das Clínicas (HC), Laboratório de Investigação Médica de Otorrinolaringologia (LIM32), São Paulo, SP, Brazil.; 2 Universidade de São Paulo (USP), Faculdade de Medicina (FM), Departamento de Otorrinolaringologia, São Paulo, SP, Brazil.; 3 Universidade de São Paulo (USP), Instituto de Biociências (IB), Centro de Pesquisa Sobre o Genoma Humano e Células-Tronco (HUG-CELL), Departamento de Genética e Biologia Evolutiva, São Paulo, SP, Brazil.

**Keywords:** SHED/c.35del, *GJB2*(Cx2*6*), *GJA1*(Cx43), cell differentiation, nonsense-mediated mRNA decay (NMD)

## Abstract

Pathogenic DNA alterations in *GJB2* are present in nearly half of non-syndromic hearing loss cases with autosomal recessive inheritance. The most frequent variant in *GJB2* causing non-syndromic hearing loss is the frameshifting c.35del. *GJB2* encodes Cx26, a protein of the connexin family that assembles hemichannels and gap junctions. The expression of paralogous proteins is believed to compensate for the loss of function of specific connexins. As Cx26 has been involved in cell differentiation in distinct tissues, we employed stem cells derived from human exfoliated deciduous teeth (SHEDs), homozygous for the c.35del variant, to assess *GJB2* roles in stem cell differentiation and the relationship between its loss of function and the expression of paralogous genes. Primary SHED cultures from patients and control individuals were compared. SHEDs from patients had significantly less *GJB2* mRNA and increased amount of *GJA1* (Cx43), but not *GJB6* (Cx30) or *GJB3* (Cx31) mRNA. In addition, they presented higher induced differentiation to adipocytes and osteocytes but lower chondrocyte differentiation. Our results suggest that *GJA1* increased expression may be involved in functional compensation for *GJB2* loss of function in human stem cells, and it may explain changes in differentiation properties observed in SHEDs with and without the c.35del variant.

## Introduction

The *GJB2* (Gap Junction Beta 2) gene (OMIM# 121011) encodes connexin (Cx) 26 (Cx26), a member of the large family of gap junction proteins (Nielsen *et al.*, 2012). Pathogenic DNA variants in the *GJB2* gene at the DFNB1 locus (13q11-12) explain nearly 50% of the cases of autosomal recessive non-syndromic deafness (Guilford *et al.*, 1994; Kenneson *et al.*, 2002). More than 300 different pathogenic DNA alterations have been reported in the *GJB2* gene (Deafness Variation Database), the majority of which leading to prelingual hearing loss with autosomal recessive inheritance pattern (Kenneson *et al.*, 2002; Snoeckx *et al.*, 2005). 

Among all known *GJB2* loss-of-function variants, c.35del (p.Gly12ValfsTer2; NC_000013.11; OMIM *121011.005) is the most frequent one, corresponding to 75% of all reported alleles with a DFNB1 pathogenic variant in European or European derived populations (Batissoco *et al.*, 2022; Lezirovitz and Mingroni-Netto, 2022). This single-nucleotide deletion occurs in one of the six repeated guanosines in the coding positions 30-35, generating a translational frameshift and a premature stop codon three bases downstream, thus potentially resulting in a truncated peptide with only 12 amino acids (Kelsell *et al.*, 1997). Although the *GJB2* gene has two exons, the Cx26-coding sequence is fully contained in its second exon. Alternative scenarios predict that the expression of the *GJB2* c.35del allele produces no protein as the mRNA may undergo decay and the truncated peptide should be additionally degraded (del Castillo and del Castillo, 2017). 

The *GJB6* gene encoding connexin 30 (Cx30) also locates at the DFNB1 locus, adjacent to the *GJB2* gene. Cx26 and Cx30 proteins have 77% similarity (Kelley *et al.*, 1999). Large deletions at the DFNB1 locus encompassing the *GJB6* gene or neighboring regions have been reported in individuals presenting with hearing loss, either in homozygosis or in trans with a single recessive mutation in the *GJB2* gene. Thus, *GJB6*-related deletions act as recessive alleles with *GJB2* variants determining hearing loss (del Castillo *et al.*, 2002; del Castillo *et al.*, 2005). In cochlea, Cx26 and Cx30 co-localize in supporting cells of the organ of Corti, in the basal cell region of the stria vascularis, and in type-1 fibrocytes of the spiral ligament (Forge *et al.*, 2003). In addition to Cx26 and Cx30, other Cxs are expressed in the cochlea, but in different regions. Cx29 (*GJC3*) is expressed in the Schwann cells wrapping the spiral ganglion neurons. Cx31 (*GJB3*) is localized in type-3 fibrocytes in the spiral ligament in cochlea lateral wall, and Cx43 (*GJA1*) is expressed in the bone of the otic capsule (Wingard and Zhao, 2015). The *Gjb1* gene coding for Cx32 is expressed in developing mouse cochlea, but no *Gjb1* transcript was observed in the adult mouse cochlea (López-Bigas *et al.*, 2002). Nevertheless, autosomal recessive or dominant non-syndromic hearing loss associated with Cx-encoding genes so far results only from variants in *GJB2*, *GJB6* and *GJB3* genes (Van Camp and Smith, 2023). 

Cxs form gap junctions, intercellular conduits for small molecules. In humans, the Cx family is composed of 21 different members with a high degree of similarity (Beyer and Berthoud, 2018). Each Cx family member can be detected in distinct cell types, and a single cell type can express different Cxs, suggesting the possibility of functional redundancy and compensation among Cxs, upon loss of expression of one of them. A Cx hemichannel (connexon) consists of six Cx units, allowing for homomeric or heteromeric hemichannels, respectively assembled by a single Cx type or more than one member of the Cx family. Two hemichannels in neighboring cells interact to form a gap junction (Račkauskas *et al.*, 2010). 

Although the *GJB2* gene is widely expressed, such as in brain, liver, uterus, testis, mammary and salivary glands, the loss-of-function c.35del variant in homozygosis results only in non-syndromic deafness and does not appear to affect other tissues (Snoeckx *et al.*, 2005; del Castillo and del Castillo, 2017). 

Different roles have been assigned to Cx26 in tissue maintenance and regeneration. Cx26 expression is upregulated during mouse keratinocyte differentiation (Lucke *et al.*, 1999), and after transection of the spinal cord of adult mice in ependymal cells lining the central canal (Fabbiani *et al.*, 2020). In human breast cancer cell lines, Cx26 reduces cell migration and promotes mesenchymal to epithelial transition (McLachlan *et al.*, 2006). Cx26 has been observed in mouse stem cells such as multipotent neocortical neural progenitor cells (Bittman and LoTurco, 1999; Ravella *et al.*, 2015) and epiblast pluripotent cells primed for differentiation (Takiguchi *et al.*, 2013; Esseltine *et al.*, 2020). Cx26 has also been functionally implicated in cells of mesenchymal origin, as in the regeneration of cochlea spiral ligament type-1 fibrocytes (Takiguchi *et al.*, 2013). 

Mesenchymal stem cells are multipotent stromal cells from connective tissue of various sources comprising bone marrow, umbilical cord, muscle and adipose tissues. These cells have the ability to differentiate *in vitro* and *in vivo* into chondrocytes, adipocytes, myoblasts and osteoblasts (Attia and Mashal, 2021). Previous studies demonstrated various subgroups of mesenchymal stem cells in human dental tissues, including mesenchymal stem cells from human exfoliated deciduous teeth (SHEDs) (Shi *et al.*, 2020). SHEDs consist of a population of postnatal stem cells with the ability to differentiate into various cell types. They offer a unique, readily accessible and non-invasive stem cell resource with limited ethical concerns (Oubenyahya, 2021). 

Despite the high prevalence of the *GJB2* c.35del variant and the fact that hearing loss is the most prevalent sensorineural disorder in humans and a major health concern worldwide (Batissoco *et al.*, 2022; Lezirovitz and Mingroni-Netto, 2022), the pathogenetic pathway from *GJB2* loss of function to hearing loss is still unclear (Smith *et al.*, 2024). Aiming at clarifying the molecular and cellular mechanisms upon loss of Cx26 in cells homozygous for the *GJB2* c.35del pathogenic variant, we established primary SHED cultures from patients and control individuals. We investigated the effects of the c.35del variant on the mesenchymal cell differentiation properties, and on the mRNA amount of the evolutionarily related genes *GJB6* (Cx30) and *GJA1* (Cx43). We present evidence that these cells have increased adipocyte and osteocyte differentiation, whereas the chondrocyte differentiation is negatively impacted. We additionally show that SHED cells with the *GJB2* c.35del variant have significantly more *GJA1* mRNA. 

## Material and Methods

### Human subjects

The research protocol was approved by the Ethics Committee of the Biosciences Institute of the University of São Paulo, São Paulo, Brazil (CONEP register 3284, Process nr 5414.7.0000.5464). Written informed consent was obtained from the legal representative(s) of all six subjects, authorizing anonymized information to be published. 

Six children were included in this study. DNA samples from peripheral blood cells of three patients had been previously analyzed for the c.35del and c.167del variants in *GJB2* and for two deletions near the *GJB6* gene (del(*GJB6*-D13S1830 and del(*GJB6*-D13S1854)), as laboratory routine in the genetic counseling service at the Human Genome and Stem Cell Research Center, Department of Genetics and Evolutionary Biology, Biosciences Institute, University of São Paulo (USP), São Paulo, Brazil (Batissoco *et al.*, 2009). 

Three unrelated children (aged 6-10 years) from this cohort of hearing-impaired patients showing the c.35del pathogenic variant in homozygosis constituted the patient (P) group. Three other unrelated children (aged 6-8 years) constituted the hearing control (C) group. DNA samples from SHEDs of three controls had been submitted to sequencing of the *GJB2* gene to confirm the absence of c.35del and of other variants in this gene. The families of hearing children who agreed to participate were interviewed regarding the existence of family history of hearing impairment, which was negative.

Two female and one male individuals constituted each, patient (P) or control (C), groups. Each child donated an exfoliated deciduous tooth, which was collected as described in Miura *et al.* (2003). The tooth was collected immediately after naturally falling out, and transported to the laboratory in DMEM/F12 (1:1), 400 U/mL penicillin, 400 µg/mL streptomycin. Upon arrival at the laboratory, the SHEDs culture protocol was promptly initiated.

### Nucleic acids sequences

Nucleic acid Reference Sequences (RefSeq) accession numbers retrieved at the National Center for Biotechnology Information (NCBI), and oligonucleotide sequences are listed on Tables S1 and S2. Amino acid sequence alignments were carried out with the CLUSTAL Omega - Multiple sequence alignment tool.

### Antibodies

Antibodies employed in this study were from Thermo Fisher Scientific (Waltham, MA). Those for flow cytometry were conjugated to phycoerythrin (PE), peridinin chlorophyll protein (PerCP), Alexa700, PerCP-Cyanine5.5 (PerCp-Cy5) or fluorescein isothiocyanate (FITC): CD29-PerCP, CD73-PE, CD90-Alexa700, CD105-PE, CD166-PE, CD31-PE, CD34-PerCP-Cy5 and CD45-FITC. Antibodies for Cx43 and alpha-tubulin were rabbit polyclonal and monoclonal antibodies, respectively.

### SHED cultures

All six different SHEDs primary cell lines were generated and assessed based on previous work (Miura *et al.*, 2003; Fanganiello *et al.*, 2015). Dental pulp fragments were retrieved with a barbed nerve broach instrument, and cells were digested in a solution of Tryple express (Thermo Fisher Scientific, Waltham, MA, USA), for one hour, at 37 ^o^C. After digestion, cells were maintained in 6-well tissue culture-treated plates containing DMEM/F12 (Thermo Fisher Scientific,Waltham, Massachusetts, USA), supplemented with 15% FBS (Fetal Bovine Serum Hyclone, Madison, WI, USA), 100 U/ml penicillin, 100 µg/ml streptomycin, 2 mM glutamine, and 2 mM non-essential amino acids (Thermo Fisher Scientific, Waltham, MA, USA). Cells were kept at 37 ^o^C in a 5% CO_2_ incubator and maintained in semi-confluence to prevent differentiation. Medium was refreshed every two days, and passages performed every four days. For that, cells were washed in PBS (Phosphate-buffered saline, pH 7.4; Thermo Fisher Scientific, Waltham, MA, USA), dissociated with Tryple Express (Thermo Fisher Scientific, Waltham, MA, USA) for seven min. and seeded in 25 cm^2^ culture flasks (Corning, St. Louis, MO, USA). The cells used in experiments were in passages 5, 6 or 7.

### DNA analysis

DNA samples were isolated from SHEDs by phenol-chloroform protocol (Batissoco *et al.*, 2009). The *GJB2* coding sequence was amplified in two PCR reactions using primer pairs 5’ ACC TGT TTT GGT GAG GTT GTG T - 3’ and 5’ ACC TTC TGG GTT TTG ATC TCC TC - 3’ for the first fragment and for the second 5’ GGA AGT TCA TCA AGG GGG AGA TA - 3 and 5’ TGA GCA CGG GTT GCC CTC ATC - 3’. The PCR fragments were submitted to Sanger sequencing using the ABI Big Dye Terminator v3.1 Cycle Sequencing Kit and the ABI 3730 DNA Analyzer (Applied Biosystems, Carlsbad, CA, USA).

### SHED characterization

Cell characterization was performed by flow cytometry and cell differentiation aiming at confirming SHEDs multipotent features. To assess the cellular phenotypes, specific cell differentiation protocols were followed by cell colorimetric assays, polymerase chain reaction (PCR) of reversely transcribed polyadenylated RNA (RT-PCR) and all in technical triplicates.

### Flow cytometry

The expression of cell surface markers was investigated in SHEDs by flow cytometry. For each antibody assayed, 1 × 10^5^ cells were initially employed. SHEDs were fixed in 4% paraformaldehyde (Electron Microscopy Sciences, Hatfield, PA) in PBS for 15 minutes at 4 ^o^C and permeabilized with the cell permeabilization kit (Thermo Fisher Scientific, Waltham, Massachusetts, USA) according to the manufacturer’s protocol. Cells were washed with PBS, blocked in 2% BSA (bovine serum albumin, Invitrogen, Carlsbad, CA, USA) for 30 minutes at room temperature, and incubated for one hour at 4 ^o^C, in the presence of the conjugated primary antibody in a 500-fold dilution in PBS, 2% BSA. Cells were washed once with PBS and resuspended in 300 μL of PBS. A minimum of 10,000 events was analyzed at FACS Aria II Flow Cytometer (BD Biosciences, Franklin Lakes, NJ, USA) and the FACS Diva software.

### Adipogenic differentiation

For the adipogenic differentiation, cells were plated in 24-well plates at 1.5 × 10^4^ cells/cm^2^ in complete medium. Twenty-four hours later, SHEDs were washed in PBS and cultivated in Stem Pro Adipogenic Differentiation Kit medium (Thermo Fisher Scientific, Waltham, Massachusetts, USA). The medium was refreshed every two days. After 15 days in culture, cells were washed three times in PBS and fixed in 4% paraformaldehyde (PFA, Electron Microscopy Sciences, Hatfield, PA) for 15 minutes. After washing twice in PBS, fixed cells were incubated with 0.16% Oil Red O (Sigma, Saint Louis, MO, USA) solution for 15 minutes. After three rinses in PBS, cells were observed and photographed under a phase-contrast inverted microscope (Axiovert 40C, Carl Zeiss, Oberkochen, Germany). The Oil Red dye was eluted from the cells using isopropanol and the absorbance at 500 nm was determined in the Epoch Microplate Spectrophotometer (BioTek, Vermont, USA). The mRNA for the adipogenic *FABP4* gene was analyzed using RT-PCR, as detailed below. 

### Osteogenic differentiation

Cells at 1.5 × 10^4^ cells/cm^2^ were plated in 24-well plates, and after 24 hours of culture in complete medium washed with PBS and cultivated with StemPro Osteogenesis Differentiation Kit medium (Thermo Fisher Scientific, Waltham, Massachusetts, USA). The medium was refreshed every two days. After three weeks in culture, the cells were washed twice with PBS and fixed in 4% paraformaldehyde (Electron Microscopy Sciences, Hatfield, PA), for 15 minutes. 

After washing the cells in PBS, they were incubated with 0.1% alizarin red solution (Sigma, USA) in Tris-HCl pH 8.3, at 37 ^o^C for 30 minutes. After two washes with PBS, cells were observed and photographed under a phase-contrast inverted microscope (Axiovert 40C, Carl Zeiss, Oberkochen, Germany). Cells stained with alizarin red were incubated for 15 minutes with 20% methanol and 10% acetic acid solution. The supernatant photometric absorbance at 450 nm was determined in the Epoch Microplate Spectrophotometer (BioTek, Vermont, USA). 

### Chondrogenic differentiation

For chondrogenic differentiation, nearly 3.5 × 10^5^ SHED cells were collected by centrifugation and seeded to culture in a conic tube with Stem Pro Chondrogenesis Differentiation Kit medium (Thermo Fisher Scientific, Waltham, Massachusetts, US). The medium was refreshed every two days. After 21 days, pelleted cells were washed twice with PBS, and fixed in 4% paraformaldehyde (Electron Microscopy Sciences, Hatfield, PA) for 15 minutes, washed in PBS and then included in tissue freezing medium (JUNG, Nussloch, DE) before submitting them to freezing at -20 ^o^C and cryo-sections (10 μm) on a cryostat (CM1850, Leica, Nussloch, Germany). The slides containing the sections were stained with 1% Alcian blue solution (Sigma, Saint Louis, MO, US) for 30 minutes and washed in 0.1 N HCl. After two washes in PBS, cells were observed and photographed under a phase-contrast inverted microscope (Axiovert 40C, Carl Zeiss, Oberkochen, Germany). The mRNA expressed by the *COL1A1*, *COL2A1* and *ACAN* genes, involved in chondrogenic differentiation, were analyzed by RT-qPCR, as described below. 

### RT-PCR and quantitative RT-PCR (RT-qPCR)

Total RNA was isolated from SHEDs of passage 7 (N = 3 per group) for the analysis of mRNA of the *GJB2* (CX26), *GJB6* (CX30), *GJB3* (CX31), *GJA1* (CX43), *GJA8* (CX50), *COL1A1, COL2A1, ACAN*, and *FABP4* genes, using the NucleoSpin RNA Plus kit (Macherey-Nagel, Düren, Germany). RNA was quantified at the Epoch Microplate Spectrophotometer (BioTek, Vermont, USA). One microgram of RNA was employed for cDNA synthesis using an oligo-dT primer and the SuperScript III kit (Thermo Fisher Scientific, Waltham, Massachusetts, USA). Primer sequences used in non-quantitative RT-PCR and quantitative RT-PCR (RT-qPCR) were designed with Primer-Blast and are presented on Table S1 and Table S2. For RT-PCR analysis of the *GJB2* gene, two different pairs of primers were designed, in both cases only cDNA was amplified. For non-quantitative PCR, the products were submitted to electrophoresis in agarose gel, stained with SYBR green. For quantitative PCR, the reactions were carried out in a Step One System, using PowerUp™ SYBR™ Green Master Mix (Thermo Scientific, Waltham, MA, USA) with each primer at 100 nM and 3 µl of cDNA (approximately 100 ng of RNA), according to the manufacturer’s protocol. The relative target gene expression was normalized by the *GAPDH* mRNA, calculated by the 2^-ΔΔCT^ based fold-change estimation (Pfaffl, 2001). For each comparison, all triplicate samples from both groups were assayed on the same plate. Samples without cDNA were negative controls for all experiments. 

### Western blotting

Undifferentiated SHED cells at approximately 90% confluence were harvested and lysed in RIPA buffer (50 mMTris-HCL, pH8, 150 mM NaCl, 50 mM sodium fluoride, 5 mM sodium orthovanadate, 2 mM EGTA, protease inhibitor cocktail (Roche, Diagnostics, Indianapolis, USA), 1% NP-40, 0.1% SDS, 0.5% sodium deoxycholate. Protein quantity was estimated using the Bradford reagent at 595nm absorbance, and 30 µg of protein in sample buffer (2% SDS, 100 mM dithiothreitol, 10% glycerol) were subjected to SDS-PAGE (10%). After electrophoresis and electro-transferring proteins to a 45-µm nitrocellulose filter (BioRad, Hercules, CA) for 16 h at 25V, transfer efficiency was verified by 1.5% Ponceau-S staining. Filter proteins were blocked for 1 h with 1% casein (Novagen, Germany), followed by 10 min in 3% hydrogen peroxide. Blots were incubated with primary antibody followed by secondary antibody, for 1 h each, at room temperature. Antibody dilutions were in 2% immunoglobulin-free bovine serum albumin (BSA, Jackson Immuno Research Laboratories, West Grove, PA) in TBS-T (20mM Tris pH 7.6, 135 mM NaCl, 0.05% Tween-20). All washes were in TBS-T. The filter was incubated in ECL^TM^ Plus substrate (GE Healthcare, Little Chalfont, UK) and exposed to Amersham Hyperfilm TM ECL film (GE Healthcare, Little Chalfont, UK). Densitometry analyses were performed using ImageJ 1.38e software (http://rsb.info.nih.gov/ij/) and Image Studio Lite (https://www.licor.com/bio/products/) to measure the intensity of bands.

### Statistical analyses

All experiments were performed in triplicates between patient and control groups. Student’s *t*-test was used for paired comparisons. Error bars in bar graphs represent standard deviation. The level of statistical significance was set at P < 0.05. Tests were performed using the GraphPad software.

## Results

### Characterization of the SHED cell lines

**Figure 1 -:**
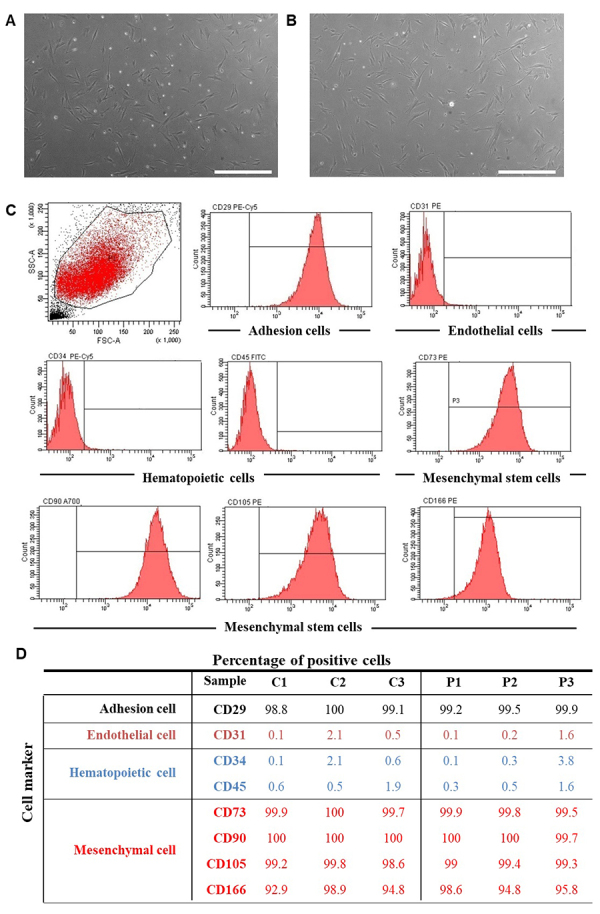
Isolation, morphological observation and flow cytometric analysis of SHEDs. (A, B) Phase-contrast images showing the fibroblast-like morphology of the in vitro expanded SHEDs from individuals without **(A)** and with c.35del **(B)** in homozygosis at P5 (scale bar: 500 µm). **(C, D)** Flow cytometric analysis of the expression of mesenchymal stem cell markers (CD73, CD90, CD105, CD166), endothelial cells markers (CD31), hematopoietic cells markers (CD34 and CD45) and cell adhesion marker (CD29). C1, C2 and C3: control group without c.35delG variant; P1, P2 and P3: group of patients with c.35del variant in homozygosis.

Six SHED cell lines were obtained from dental pulp tissue clumps after nearly 15 to 20 days in culture. They exhibited typical fibroblast-like appearance as spindle-shaped cells with small size and low granularity (Figures 1A ,B). 

The six lineages of isolated SHEDs were analyzed at passage 5 by flow cytometry to determine the expression of cell surface markers (Figure 1C ), specifically for mesenchymal stem cells (CD73, CD90, CD105, CD166), endothelial cells (CD31), hematopoietic cells (CD34 and CD45) and cell adhesion (CD29). The analysis indicated that all SHEDs highly expressed CD29 (99.55±0.45%), CD73 (99.75±0.25%), CD90 (99.85±0.15%), CD105 (99.2±0.6%) and CD166 (95.9±3.0%) markers, while they were nearly negative for the hematopoietic (CD34, 1.95±1.85%; and CD45, 1.1±0.8%) or endothelial (CD31, 1.1 ±1.0%) cell markers (Figure 1D ). Summing up, all six cultures expressed characteristic markers of cells of mesenchymal origin and were negative for the expression of markers of hematopoietic and endothelial cells (Figure 1), as expected (Miura *et al.*, 2003; Fanganiello *et al.*, 2015). 

### *GJB2 c*.35del SHEDs have altered differentiation properties

**Figure 2 -:**
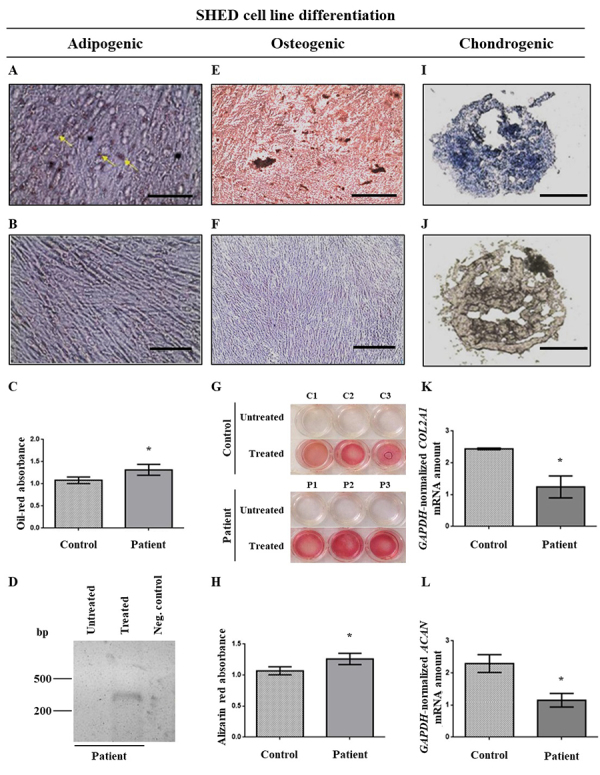
Analysis of adipogenic (A-D), osteogenic (E-H) and chondrogenic (I-L) differentiation of SHEDs. (A) Following 14 days of culturing of cells under *in vitro* treatment with adipogenic medium, the SHEDs presented fat deposits seen after Oil-Red staining. The yellow arrows indicate fat deposits, evidenced by the brown color. In **(B)**, non-treated SHEDs. Scale bar = 100 µm. **(C)** Quantification of adipogenic differentiation by Oil-Red absorbance at 500nm from samples as shown in (A) and (B). SHEDs with c.35del had more lipid laden cells (Student´s *t*-test, P˂0.05; n = 3). **(D)** Agarose gel after electrophoresis of *FABP4* transcript RT-PCR products of SHED cDNA. Qualitative data are shown as presence or absence of DNA band. DNA size ladder: 100pb (Invitrogen, Carlsbad, CA, USA). In **(E)**, **(F)** and **(G)**, representative matrix mineralization assayed by Alizarin Red staining after 21 days of *in vitro* treatment with osteogenic medium. After staining with Alizarin Red, calcium deposits are stained in red in **(E)** and in non-treated SHEDs **(F)**. Scale bar = 400 µm. In **(G)** an image of cells treated and not treated with osteogenic differentiation medium and stained with Alizarin Red. **(H)** Quantification of Alizarin Red staining by absorbance at 450 nm of samples as shown in (E) and (F). There were more SHEDs from the c.35del group with calcium deposits (Student´s *t*-test, P˂0.05; n=3). (**I**) Representative pellet of SHEDs after 21 days of *in vitro* treatment with chondrogenic medium stained with Alcian Blue. **(J)** No staining observed for untreated SHED cells. Scale bar = 100 µm. **(K-L)** Quantification of chondrogenic differentiation by RT-qPCR analysis of *COL2A1* and *ACAN* genes, normalized by *GAPDH* RT-qPCR, respectively. The mRNA quantity of the *COL2A1* **(K)** and *ACAN* **(L)** genes was significantly higher (P˂0.05; n = 3) in the group of individuals without the c.35del variant. Values for comparison in statistical analyses were obtained by the 2-∆∆Ct approach. The bars represent the means with the standard deviations of the relative gene expression values of each group.

The six undifferentiated SHED cell lines were analyzed for their adipogenic, osteogenic and chondrogenic differentiation potentials. For each cell line, untreated samples were maintained in complete medium. After two weeks of the *in vitro* treatment with the adipogenic medium, numerous lipid vacuoles were observed in both SHED groups as stained by Oil Red (Figure 2A , arrows), when compared to the untreated control (Figure 2B ). In order to investigate whether the differentiation potential of the cells was influenced by the *GJB2* c.35del variant, SHEDs treated for adipogenic differentiation were incubated with isopropanol to remove the previously incorporated Oil Red stain, and the supernatant had its optical density measured at the 500-nm absorbance. Differentiated SHEDs from the c.35del group showed intense reaction with Oil-Red with higher absorbance when compared to the control group (Figure 2C ; P=0.048; n=3). This result indicated that SHEDs were able to differentiate into adipocytes under the applied treatment conditions, and more cells in the patient group succeeded in adipogenic differentiation. Moreover, SHEDs treated *in vitro* for adipogenic differentiation expressed the *FABP4* (fatty acid binding protein 4) gene, encoding an adipocyte cytoplasmic protein marker, while those not treated *in vitro* for differentiation did not present the mRNA (Figure 2D ). 

The osteogenic treatment lasted for 21 days and cells were then incubated with alizarin red, which stains calcium deposits, an effect of osteocyte differentiation. We observed a large number of calcified nodules in the six lineages submitted to osteogenic differentiation, but not in the untreated groups (Figure 2E ,F). Alizarin red-stained cells were incubated with methanol/acetic acid, and the supernatant had its optical density measured at the wavelength of 450 nm. The c.35del group showed a higher optical density at 450 nm when compared to the control group (Figure 2G ,H; p=0.041, n=3), allowing to infer increased osteogenic differentiation. 

To assess the chondrogenic differentiation capacity, SHEDs were cultured in a specific treatment medium for three weeks. After fixation and cryosectioning of the pelleted cells, Alcian blue was used to detect extracellular matrix proteoglycans, a chondrogenesis marker. Positive staining was observed in the six SHED lineages after chondrogenic differentiation treatment (Figure 2I ). Control samples of each of the six cell lines, which were maintained in complete culture medium for 21 days, were negative (Figure 2J ). As the chondrogenic assay does not allow reporting a measurable variable, quantitative RT-PCR (RT-qPCR) was used for the analysis of transcripts of three genes with characteristic expression in chondrocytes (*COL1A1*, *COL2A1* and *ACAN*), and the results of SHEDs of individuals with and without the c.35del variant were compared. The control group had higher *COL2A1* (Figure 2K ; P=0.039, n=3) and *ACAN* (Figure 2L ; P=0.044, n=3) mRNA quantities than the patient group. The amount of mRNA expressed by the *COL1A1* gene was not altered when the c.35del group and the control group were compared (data not shown). The increased expression of chondrocyte markers in the control group indicates decreased chondrogenic differentiation of SHEDs with the c.35del allele in homozygosis.

### Expression of the *GJB2* (Cx26) gene

**Figure 3 -:**
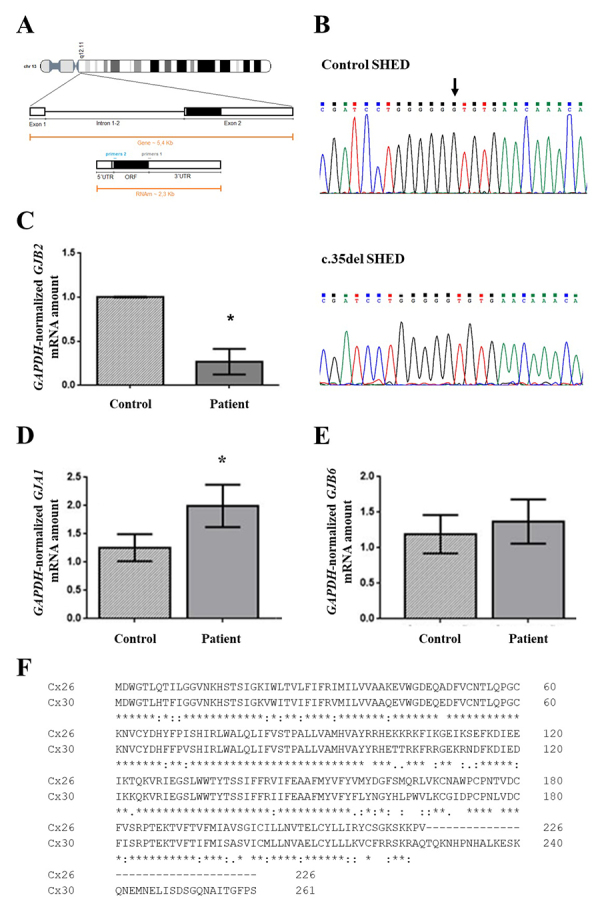
*GJB2, GJA1* and *GJB6* analysis. **(A)** Diagram of the *GJB2* gene with two exons and one intron, indicating the location of the coding sequence and 3’ UTR on the second exon. (**B)** Result of genomic DNA sequencing of undifferentiated SHED from a hearing individual (without c.35del) and from an individual with homozygous c.35del. The arrow indicates the position of the pathogenic variant**. (C, D and E)** Analysis of *GJB2* **(C)** *GJA1* **(D)** and *GJB6* **(E)** gene expression in SHEDs at P7. RT-qPCR results of relative mRNA amount normalized to *GAPDH* in P7 SHEDs without (control) and with c.35del (Patient). **(F)** Alignment of Cx26 (226 amino acids) and Cx30 (261 amino acids) sequences. (*) indicates identical residues. Significant P-values are indicated according to Student´s *t*-test results. The bars represent the means with the standard deviations of the relative gene expression values of each group. N=3 for all comparisons. (*) P <0.05.

Sanger sequencing of the *GJB2* coding sequence confirmed the c.35del variant in homozygosis in the three SHEDs of the patients (P) group. In hearing controls (C), no variant was identified in the *GJB2* gene (Figures 3A  and 3B). RT-qPCR disclosed the *GJB2* mRNA in undifferentiated SHEDs from both groups. SHEDs of individuals with the c.35del variant had less *GJB2* mRNA than those from individuals without the variant (Figure 3C ; P =0.007, n = 3).

### *GJA1*, *GJA8*, *GJB3* and *GJB6* gene expression in SHED cell lines

**Figure 4 -:**
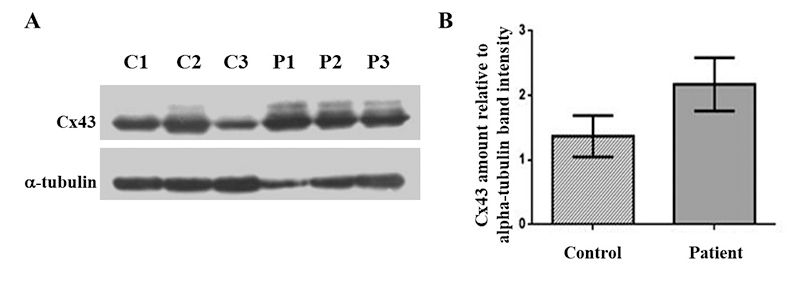
Analysis of CX43/*GJA1* expression in undifferentiated SHEDs at P7. Immunoblotting of cell lysate supernatant of control (C1-C3) and patient (P1-P3). SHED cells are probed with the anti-Cx43 antibody. The immunoblotting results show Cx43 in all samples (A). The Cx43 amount normalized by that of α-tubulin band intensity disclosed a trend to increase in patient samples (P=0.056; B). Significant P-values are indicated according to Student´s *t*-test results. The bars represent the means with the standard deviations of the relative gene expression values of each group. N=3 for all comparisons. (*) P <0.05.

The expression of paralogous connexin-encoding genes with biological relevance for the cochlea was assessed by RT-PCR of undifferentiated SHEDs cDNA. The investigated genes were *GJA1*, *GJB3* and *GJB6*, respectively encoding Cx43, Cx31 and Cx30, as well as a putative negative control (*GJA8* gene, coding for Cx50). RT-PCR amplification was not observed for the *GJA8* or *GJB3* mRNA of SHEDs from either group (data not shown). As we observed on agarose gel the RT-PCR products of *GJA1* and *GJB6* of both control and patient SHED cDNA (data not shown), we conducted RT-qPCR to compare the mRNA amount between groups. SHEDs from the c.35del group had more *GJA1* mRNA when compared to the control group (Figure 3D ; P = 0.044, n = 3). The quantities of the mRNA of the *GJB6* gene did not differ between groups (Figure 3E ; P˃0.05; n=3). As the proteins encoded by the *GJB2* (Cx26) and *GJB6* (Cx30) genes are highly similar and SHED cells from control individuals co-express them (Figure 3F ), it was not possible to assess their quantities (data not shown). On the other hand, western blotting confirmed the expression of Cx43 in the six established cell lines (Figure 4A ). The semi-quantitative analysis of Cx43 band densitometry, normalized by the alpha-tubulin band intensity, disclosed a trend to increase in the Cx43 amount in SHED lines of individuals with the c.35delG variant (P = 0.056; n = 3) (Figure 4B ).

## Discussion

In this study, we established SHED cell lines from control individuals and hearing-impaired patients with the *GJB2* c.35del variant in homozygosis. We show altered cell differentiation properties and upregulated expression of the paralogous *GJA1* gene encoding Cx43, upon reduction of *GJB2* mRNA in SHED cells. 

Pluripotent and multipotent stem cells (SC) from patients with genetic diseases present great potential to recapitulate at the cellular level phenotypic changes elicited by pathogenic DNA variants, thus allowing for investigation of pathophysiological mechanisms and possibly testing of drugs for future therapeutic interventions (Zakrzewski *et al.*, 2019). The *GJB2* gene is expressed in human embryonic stem cells (ESCs), induced pluripotent stem cells (IPSCs), and derived embryonic bodies and neural cells (Jin *et al.*, 2015). IPSCs obtained from human individuals with *GJB2* c.109G>A/(p.Val37Ile) differentiated into the major cells of the three germ layers (Lu *et al.*, 2020; Fukunaga *et al.*, 2021; Colbert *et al.*, 2022). IPSCs harboring a frameshifting pathogenic variant 200 nucleotides downstream of c.35del (*GJB2*, c.235del, p.Leu79CysfsTer3) were able to differentiate into neural progenitor cells and neurons *in vitro* and overexpressed the *GJB1* mRNA (Degen *et al.*, 2011; Jin *et al.*, 2015). Moreover, it has been shown that IPSCs homozygous for the c.235del allele had deficient gap junction activity assessed by dye transfer between cells (Fukunaga *et al.*, 2021). These works demonstrate the importance of studying *GJB2* variants in human-derived SCs at distinct differentiating states and cell types.

The human oral cavity is home to multipotent mesenchymal SCs from various niches, including dental pulp, periodontal ligament, dental follicle precursor cells, apical papilla and gingiva. Among them, SCs obtained from human dental pulp can be easily isolated from SHEDs or extracted permanent teeth, with lesser ethical restraints than SCs from other biological sources (Shi *et al.*, 2020; Oubenyahya, 2021). SHEDs exhibit high proliferation rate and have similar properties to other multipotent mesenchymal SCs (Attia and Mashal, 2021), such as the ability to rapidly adhere to plastic surfaces and expression of specific cell surface marker (CD73, CD90, CD105, CD166) and transcription factors of undifferentiated cells (*OCT4*, *SOX2*, and *NANOG*). In addition, as mesenchymal SCs, SHEDs can differentiate into cell lines of mesenchymal origin as osteocytes, chondrocytes, and adipocytes (Miura *et al.*, 2003; Dominici *et al.*, 2006; Govindasamy *et al.*, 2010). The mesenchymal SCs nature of the six SHED cell lines established in this study was confirmed by flow cytometry (McLachlan *et al.*, 2006). Cells of passage 5 were positive for characteristic markers of mesenchymal cells and cell adhesion, and negative for markers of hematopoietic or endothelial cells (Figure 1). At flow cytometry, no phenotypic difference was apparently observed between groups of undifferentiated SHEDs with and without the *GJB2* c.35del variant. Moreover, the six SHEDs lines had the properties to undergo osteogenic, chondrogenic and adipogenic differentiation (Figure 2).

The *GJB2* coding sequence is fully contained within its last exon (exon 2). The c.35del variant causes a +1 frameshift in the *GJB2* translation reading frame, leading to an amino acid change at position 12 (p.Gly12ValfsTer2) immediately followed by a premature termination codon (PTC). Hence, the predicted truncated peptide has 12 amino acids instead of the expected 226 residues (Smith *et al.*, 2024). It is largely expected that PTC-containing mRNAs and truncated proteins undergo decay. Here we observed a decrease in the *GJB2* mRNA amount in undifferentiated SHED cells homozygous for the c.35del variant as compared to the control group (Figure 3C ). This is consistent with increased susceptibility to degradation of the frameshifted transcript, since no transcription rate difference is anticipated between the wild-type and c.35del alleles.

The nonsense-mediated mRNA decay (NMD) is a molecular pathway that targets mRNAs with PTC for degradation, down-regulating abnormal transcripts. NMD relies on the identification of the PTC by the RNA helicase and ATPase UPF1 (Upstream Frameshift Protein 1) during a pioneer translation round of the mRNA upon its arrival in the cytoplasm (Kurosaki *et al.*, 2019). UPF1 phosphorylation and accessory proteins are crucial to elicit the decay of PTC-containing mRNAs, in particular UPF1 interaction with UPF2, a regulator of NMD (Kashima *et al.*, 2006; Kurosaki *et al.*, 2014).

Although the NMD mechanism is evolutionarily conserved in eukaryotes from yeast to mammals, different molecular complexes have evolved to discriminate between the wild-type termination codon and upstream PTCs. Upon splicing of the primary transcript of multi-exon genes, the exon-junction protein complex (EJC) deposits at nearly 24 nucleotides 5’ to each junction between exons in the mature mRNA. Once the processed mRNA is exported from the nucleus to the cytoplasm, UPF2 promptly associates with EJCs (Kurosaki *et al.*, 2019). After removal of EJCs from the mRNA in the pioneer translation round by the ribosome, PTCs located at least 50-55 nucleotides upstream of an EJC generally trigger NMD by allowing for direct binding between the downstream EJC, UPF2 and UPF1 (Kurosaki *et al.*, 2019). However, many eukaryotic genes have the full coding sequence continuous with the 3’ untranslated (3’ UTR) region in a single exon, as is the case of the *GJB2* gene. Hence, PTCs are not expected to elicit NMD of their transcripts by the mechanism described above. Since we found evidence that the mRNAs produced by the *GJB2* c.35del allele were downregulated, an additional model must be considered to trigger their degradation. 

Fail-safe NMD, also known as 3’ UTR EJC-independent NMD, is a non-canonical mammalian pathway that elicits NMD in the absence of a downstream exon-exon junction, on the condition that at least one exon-exon junction lies upstream of a PTC (Matsuda *et al.*, 2007). When the *GJB2* primary transcript undergoes splicing, intron 1 is removed, and one EJC is expected to be placed upstream of the junction between exons 1 and 2 on the mature mRNA. The human *GJB2* exon 2 contains the full Cx26-encoding sequence (681 bp) and 1,431 bp of 3’ UTR. Hence, the c.35del-associated PTC is expected to extend the *GJB2* 3’ UTR in 641 nucleotides. Fail-safe NMD relies on long 3’ UTRs of mRNAs with PTC, if there are no specific NMD-inhibiting RNA structures within it (Raimondeau *et al.*, 2018). The raise in the 3’ UTR length increases the distance between the translation termination codon and the mRNA poly-A tail coated by poly (A) binding protein cytoplasmic 1 (PABPC1) (Behm-Ansmant *et al.*, 2007), a known co-activator of translation termination through its association with eukaryotic release factor 3A (eRF3A) (Hoshino *et al.*, 1999; Uchida *et al.*, 2002). Although the fail-safe NMD mechanism is not fully understood, the longer 3’ UTR should impair efficient interaction between PABPC1 and eRF3A, thus attenuating translation termination and activating the mRNA decay. The fail-safe NMD hypothesis appears more likely to explain the decay of the *GJB2* mRNA expressed by the c.35del allele observed in this study. However, the co-translational degradation of mRNA producing truncated peptides as well as other RNA degradation pathways such as the nuclear exosome should not be dismissed (Gockert *et al.*, 2022). 

The overexpression of Cx26 in the cochlea of the *Gjb6* (Cx30) knockout mouse harboring extra copies of the *Gjb2* gene functionally restored hearing and organ of Corti hair cells (Ahmad *et al.*, 2007; Boulay *et al.*, 2013). By contrast, although endogenous Cx30 is raised in the cochlea of *Gjb2* knockout mice, it does not rescue the hearing function in the absence of Cx26 (Lee *et al.*, 2015). This indicates that Cx26 may compensate for the lack of Cx30 in the cochlea, but the reverse situation is limited (Mammano, 2019). On the other hand, increased expression of the *Gjb1* (Cx32) mRNA has been described in the cochlea of the *Gjb2* knockout mice, although Cx32 is not physiologically detected in adult cochlea that expresses endogenous wild-type Cx26 (Degen *et al.*, 2011). While it seems logical that paralogous Cxs should functionally compensate for the lack of Cx26 in non-cochlear tissues, the cell phenotype and the profile of paralogous Cx expression have not been studied in human cells that lack Cx26 due to *GJB2* loss-of-function DNA variants (Jin *et al.*, 2015; Fukunaga *et al.*, 2020; Lu *et al.*, 2020; Fukunaga *et al.*, 2021; Colbert *et al.*, 2022). Here we found that *GJA1* mRNA is upregulated in SHEDs with reduced *GJB2* mRNA carrying the Cx26-truncating c.35delG variant. We hypothesize that this increased expression may result from a cellular response to the reduction of *GJB2* mRNA in SHEDs with c.35del pathogenic variant in homozygosis. It led us to assume that the increased expression of *GJA1* transcript is somehow altering the differentiation capacity of cells with c.35del in a probable compensation mechanism that has not been previously described. However, the regulatory mechanisms of SHED with down-regulated *GJB2* gene expression that elicit elevation of *GJA1* mRNA synthesis or impair its degradation remain to be clarified.

SHEDs of patients with the c.35del variant had greater adipogenic and osteogenic differentiation and lower chondrogenic differentiation capacities in relation to SHEDs of individuals without this variant. Furthermore, patients’ SHEDs had increased amounts of *GJA1* mRNA and a trend to enhance Cx43, the encoded protein, as verified by RT-qPCR (Figure 3D ) and Western blot (Figure 4), respectively. It has been observed that pre-adipocyte 3T3-L1 cells lose the ability to form gap junctions during late adipogenesis (Azarnia and Russell, 1985; Yanagiya *et al.*, 2007). Blocking gap junctions formed by Cx43 during the early but not late stages of differentiation of 3T3-L1 cells inhibits adipogenic differentiation (Yanagiya *et al.*, 2007). Moreover, murine bone marrow-derived stromal H-1/A cells induced to differentiate into adipocytes down-regulate the expression of Cx43 at a later stage (Umezawa and Hata, 1992), and Cx43 degradation is necessary for successful terminal adipogenic differentiation (Yeganeh *et al.*, 2012). On the other hand, it has been demonstrated that Cx43 is necessary for mesenchymal cell senescence, but not for adipogenic differentiation of human mesenchymal SC (Shao *et al.*, 2019). Thus, greater adipogenic differentiation may be related to the increased amounts of *GJA1* mRNA observed in this study.

Although the studies on the roles of Cxs in the chondrogenic differentiation are limited (Schrobback *et al.*, 2015), the expression of connexins is more widely studied in the osteogenic differentiation. Pathogenic variants in the *GJA1* gene cause oculodentodigital dysplasia (Paznekas *et al.*, 2003). Knockout mice for the *Gja1* gene show hypomineralization of craniofacial bones and delayed ossification of the appendicular skeleton (Lecanda *et al.*, 2000). Different *in vitro* studies with osteoblasts have shown that hemichannels formed by Cx43 interact with different molecules to modulate cell signaling. In response to bisphosphonates, Cx43 interacts with the Src kinase, which, in turn, activates the ERK complex in the cell proliferation pathway, inhibiting apoptosis (Plotkin *et al.*, 2002). Cx43 can also interact with β-arrestin in response to parathyroid hormone, leading to cell proliferation and differentiation (Bivi *et al.*, 2011), and finally it can interact with Cδ protein kinase in response to growth factor FGF2 (Niger *et al.*, 2010). During muscle development, overexpression of Cx43 was observed to increase myoblast skeletal differentiation and myotube formation (Merrifield and Laird, 2016). Altogether, the literature suggests that our data on differentiation of SHED with *GJB2* c.35del variant could be explained by the increased expression of the *GJA1* gene in these cells. This effect could relate in part to their role in gap junction formation, but it seems to be mainly linked to the role that hemichannels and membrane connexins play in cell signaling pathways that stimulate differentiation. Finally, Cx26 has been implicated in cell proliferation control in distinct cell types, such as SCs and epithelial cells as well as in ependymal cells of the spinal central canal in neonate mice and, upon injury of the spinal cord in the adult mouse (Bittman and LoTurco, 1999; Lucke *et al.*, 1999; McLachlan *et al.*, 2006; Ravella *et al.*, 2015; Esseltine *et al.*, 2020; Fabbiani *et al.*, 2020). Therefore, the loss of *GJB2* function in SCs could down-regulate their proliferation and self-renewal that, together with the endogenous upregulated expression of Cx43, would accelerate the differentiation of these mesenchymal cells. In this scenario, labelling dyes assessing permeation of Cx43 gap junctions as well as the specific blockage of hemichannels would be useful to dissect the possible roles of Cx43 overexpression upon loss of function of Cx26.

## Supplementary material

The following online material is available for this article:

Table S1 - Oligonucleotides used as primers in RT-PCR.

Table S2 - Oligonucleotides used as primers in RT-qPCR analysis.
