# Supplementary material for: GJB2 c.35del variant up-regulates GJA1 gene expression and affects differentiation of human stem cells
Source: Genet Mol Biol. 2024 Apr 15;47(2):e20230170. doi: 10.1590/1678-4685-GMB-2023-0170 (PMC11021044; doi:10.1590/1678-4685-GMB-2023-0170)
Supplement: Table S1 - [file 1415-4757-GMB-47-02-e20230170-s1.pdf]

Genetics and Molecular Biology

https://doi.org/10.1590/1678-4685-GMB-2023-0170

Supplementary Material to "*GJB2 c*.35del variant up-regulates *GJA1* gene expression and affects differentiation of human stem cells"

Table 1 - Oligonucleotides used as primers in RT-PCR.

Gene	***RefSeq* (NCBI)**	***Primers Forward and Reverse (5´- 3´)***	***Amplicons (pb)***
GJB2<br>*(Pair 1)*	NM_004004.6	GTTTAACGCATTGCCCAGTT<br>GGCCTACAGGGGTTTCAAAT	150
GJB2<br>*(Pair2)*	NM_004004.6	GACGCAGAGCAAACCGCCCAGAGTAG<br>ATAGCCGGATGTGGGAGATGGGGAAGT	258
GJB3	NM_024009.3	TGGGCCCTGCAGCTCATCTTCGTCACA<br>CTTCTCGGTAGGTCGGGCAATGTAGCAGT	324
GJB6	NM_001110219.3	GGGCCCTCCAGCTGATCTTCGTCTCC<br>TTCTCTCCTTTGGGCATGATTGGGGTGAT	490
GJA1	NM_000165.5	GGCGGGAAGCACCATCTCTAACTCCCATGC<br>TCTCTTATCCCCTCCCTCTCCACCCATCTACCC	390
GJA8	NM_030772.5	TCCCGGGGCTACCAAGAGACACTG<br>CCTGGCTCCGGCTGCTGGCTTTGCTTAG	345
FABP4	NM_001442.3	AAGAAGTAGGAGTGGGCTTTG<br>CCACCACCAGTTTATCATCCT	285
