# Supplementary material for: GJB2 c.35del variant up-regulates GJA1 gene expression and affects differentiation of human stem cells
Source: Genet Mol Biol. 2024 Apr 15;47(2):e20230170. doi: 10.1590/1678-4685-GMB-2023-0170 (PMC11021044; doi:10.1590/1678-4685-GMB-2023-0170)
Supplement: Table S2 - [file 1415-4757-GMB-47-02-e20230170-s2.pdf]

Genetics and Molecular Biology

https://doi.org/10.1590/1678-4685-GMB-2023-0170

Supplementary Material to "*GJB2 c*.35del variant up-regulates *GJA1* gene expression and affects differentiation of human stem cells"

Table 2 - Oligonucleotides used as primers in RT-qPCR analysis**.**

Gene	***RefSeq* (NCBI)**	***Primers Forward and Reverse (5´- 3´)***
GJB2	NM_004004.6	GAGAGAGAGA CCAAC<br>GGTGGAGTGTTTGTTCACACC
GJB6	NM_001110219.3	AGGCACTCCAGTGGGGTAGGA<br>GTGCAGCGTCCCCCAATCCA
GJA1	NM_000165.5	GGTCTGAGTGCCTGAACTTGCCT<br>AGCCACACCTTCCCTCCAGCA
COL1A1	NM_000088.4	CAGCCGCTTCACCTACAGC<br>TTTTGTATTCAATCACTGTCTTGCC
COL2A1	NM_001844.5	GGCAATAGCAGGTTCACGTACA<br>CGATAACAGTCTTGCCCCACTT
ACAN	NM_001135.4	TGCATTCCACGAAGCTAACCTT<br>GACGCCTCGCCTTCTTGAA
GAPDH	NM_002046.7	AGAAAAACCTGCCAAATATGATGAC<br>TGGGTGTCGCTGTTGAAGTC
